# Perceived Factors Contributing to the Subjective Wellbeing of Undergraduate Engineering Students: An Exploratory Study

**DOI:** 10.3390/ijerph192316284

**Published:** 2022-12-05

**Authors:** Muhammad Asghar, Angela Minichiello, Assad Iqbal

**Affiliations:** 1 Engineering Education Department, Utah State University, Logan, UT 84322, USA; 2 Engineering Education Systems & Design Department, Arizona State University, Mesa, AZ 85212, USA

**Keywords:** subjective wellbeing, mental health, engineering education, undergraduates

## Abstract

Engineering education is perceived to be a tough field of study with detrimental effects on the mental health of undergraduate engineering students. High levels of anxiety and depression are reported among this population. Overall, mental health research is often biased toward looking at mental health from a deficit perspective and investigating mental health as a negative phenomenon. This trend also persists in engineering education research. The purpose of this exploratory study, therefore, is to investigate the condition of subjective wellbeing (SWB) of undergraduate engineering students to understand the factors that they perceive as positively contributing to their overall wellbeing in an engineering college environment. Qualitative data from eight undergraduate engineering students interviewed in fall 2021 in the college of engineering at a land grant public institution in the western USA were thematically analyzed. The resulting 11 themes were then re-grouped and conceptualized into 7 factors (faculty support, learning experiences, support environment, financial support, engineering practice opportunities, task organization, and task orientation) for clear mapping, understanding, and explanation. The outcomes from this research have positive implications for the SWB of undergraduate engineering students, with support from their educational institutions.

## 1. Introduction and Background

Mental health issues are prevalent across all engineering education disciplines [1]. Undergraduate engineering students report high levels of anxiety and depression [1,2,3]. Students report that they perceive the engineering college environment to be unsupportive and challenging to the point of being stressful [4]. Other studies point to stress inherent in engineering education environments by reporting on high levels of stress among undergraduate engineering students [3,5]. Most recently, the COVID-19 pandemic has been shown to negatively affect the overall adverse mental health condition of engineering students [6,7,8,9].

Educational institutions play a vital role in preventing mental health problems in students and resolving such problems once they occur. They can support students to efficiently confront their collegiate challenges with a positive mindset [10] and potentially avoid consequential mental health problems. The focus of psychological counseling services, however, is usually on curing mental health issues once they occur, with less care for preventive measures [11]. This may be due to a historical focus in mental health practice and research that associates mental health with mental illness and nothing more [12]. As a result, a research gap exists in mental health research as it is often biased toward looking at mental health from a deficit perspective and investigating mental health as a negative phenomenon [13]. This present research study aims to contribute to filling this research gap in mental health and wellbeing research in engineering education by investigating positive influences on the mental health and wellbeing of engineering undergraduates from an anti-deficit perspective. 

Our focus in this research is to respond to recent calls by engineering education researchers who want to change engineering culture from one of stress and survival to one of wellbeing and thriving [14,15] and who advocate for balancing “mental health” research in engineering education, by considering both positive and negative mental health attributes [16]. To do so, we purposefully investigated positive factors that may be present in an engineering college environment and contribute to the subjective wellbeing (SWB) of the undergraduate engineering students enrolled there. SWB is a concept based in positive psychology. In the following, we provide a brief overview of positive psychology and then describe SWB.

### 1.1. Positive Psychology

The realization of the need to improve the quality of human life can be traced back to Aristotle, who was interested in investigating *eudaimonia* (the highest human good) [13]. In the 20th century, sociologist Aaron Antonovsky and psychologists Carl Rogers and Abraham Maslow shared the same interests [13] (p. 3). Maslow coined the term positive psychology to signify the importance of accurately understanding the human potential, contrary to the traditional concern of psychology for human limitations and dysfunctions around the middle of the 20th century [17]. As a field, positive psychology was founded by psychologist Martin Seligman in the last decade of the 20th century. Seligman and Csikszentmihalyi [18] define the field of positive psychology as:

*“The field of positive psychology at the subjective level is about valued subjective experiences: well-being, contentment, and satisfaction (in the past); hope and optimism (for the future); and flow and happiness (in the present). At the individual level, it is about positive individual traits: the capacity for love and vocation, courage, interpersonal skill, aesthetic sensibility, perseverance, forgiveness, originality, future-mindedness, spirituality, high talent, and wisdom. At the group level, it is about the civic virtues and the institutions that move individuals toward better citizenship: responsibility, nurturance, altruism, civility, moderation, tolerance, and work ethic”*

(p. 5).

Today, the field of positive psychology is prominent in both research and practice that center on happiness and wellbeing [19] and is considered a vehicle for positive social change [20]. Positive psychology provided a foundation for the conception of this study and was vital to its successful execution of it.

The work of Diener et al. [21] provided a basis for the conceptualization of SWB. They termed it as a broader phenomenon that characterizes people’s perceptions of their emotions, domain satisfaction, and overall life satisfaction. In contrast to SWB, which is a subjective indicator of peoples’ wellbeing based on their personal account, objective wellbeing is socially informed based on societal indicators such as economic prosperity and the smooth functioning of society [21]. Seligman, the founder of positive psychology, has associated SWB with feelings of happiness “defined by high ends of measures of several psychological states” [22]. These states can exist on personal, emotional, or social domains [23]. Yıldırım [24] reported an increase in SWB to be positively correlated with life satisfaction and positive feelings as compared to negative feelings.

### 1.2. Purpose

The purpose of this exploratory study is to investigate the condition of subjective wellbeing (SWB) of undergraduate engineering students to understand the factors that they perceive as positively contributing to their overall wellbeing. The present study was conducted in advance of a larger mixed-methods research project. The current study was guided by the following research question:

RQ: What and how do different factors positively contribute to the subjective wellbeing of undergraduate engineering students?

Qualitative data were generated by interviewing undergraduate engineering students about the condition of their subjective wellbeing (SWB). The data were then qualitatively analyzed using thematic analysis to develop open-ended survey questions for use in the main, mixed-methods dissertation study. 

### 1.3. Conceptual Framework and Theoretical Background

Conceptual frameworks are used in empirical research to connect gaps in the existing literature with relevant theories [25] and, hence, advance scientific knowledge [26]. Researchers may use theoretical frameworks, which employ an existing theory, or they may use multiple constructs from a number of theories and develop their own conceptual framework to serve the specific purpose(s) of their research [27]. Despite their usefulness, both theoretical frameworks and conceptual frameworks have been historically underutilized in engineering education research (EER) [28,29]. For this study, we employed an existing conceptual framework developed from the constructs of college student covitality and based in positive psychology [23,30]. 

#### 1.3.1. Covitality and Subjective Wellbeing

Covitality is a contrasting concept to comorbidity [31]. Thus, while comorbidity is the co-existence of multiple psychological problems [32], covitality is the co-existence of multiple positive psychological components (cognitive, emotional, social, and behavioral) [31]. Renshaw and Bolognino [30] redefined covitality as:

*“An individual’s cumulative subjective wellbeing, which, “at the most basic level, consists of a combination of emotional, cognitive, social, and behavioral components—how people feel, think, relate, and act—that are either valued for their own sake (e.g., life satisfaction and connectedness) or because they function to attain things that are valued for their own sake (e.g., self-efficacy and perseverance), or possibly both (e.g., positive emotions)”*

(p. 464).

The conceptual framework of college student covitality developed by Renshaw and Bolognino [30] is based on the interplay of four college-specific constructs, academic satisfaction, academic efficacy, school connectedness, and college gratitude, and their contributions to college student SWB. This conceptual framework was operationalized to (a) construct and validate the College Student Subjective Wellbeing Questionnaire (CSSWQ) [30] and (b) update and revalidate the CSSWQ [23]. The four constructs are active in three (cognitive, social, and emotional) college-specific domains. Academic satisfaction and academic efficacy are situated in the cognitive domain, while school-relatedness exists in the social domain, and college gratitude exists in the emotional domain. Renshaw and Bolognino [30] theorized the existence of these college-specific domain constructs from general positive psychological constructs. Academic satisfaction is theorized from life satisfaction, academic efficacy from self-efficacy, school connectedness from social connectedness, and college gratitude from gratitude. The description of the four college-specific constructs and the corresponding theories in which their foundations reside are explained in detail in the following discussion.

#### 1.3.2. Academic Satisfaction

Academic satisfaction is derived from life satisfaction. Life satisfaction is defined as the acceptance of the surrounding circumstances and consideration of them as positive actors toward the achievement of set goals [33,34]. Similarly, academic satisfaction is defined as the “enjoyment of one’s role or experiences as a student” [35]. In other words, academic satisfaction is how people subjectively assess the quality of their lives under given circumstances, be it in social or academic contexts. A prevailing sense of life satisfaction not only positively influences professional lives but also has positive outcomes for social relationships and personal states of mind. For example, it increases productivity at work, contributes toward the formatting of a stronger support network, and decreases the level of stress [33,36]. Life satisfaction in general has a positive effect on the quality of college life [37]. Academic satisfaction is related weakly to academic performance and moderately to strongly with the students’ intentions to remain at their educational institution or their retention intention [38].

#### 1.3.3. Academic Efficacy

Academic efficacy is defined as “a person’s belief in his or her capability to successfully perform a particular task” by Albert Bandura, the founder of social learning theory [39]. A person’s belief in their abilities protects against feelings of anxiety and depression [40]. In academic contexts, academic efficacy has been associated with academic achievement [41]. Honicke and Broadbent [42] suggested a moderate correlation between academic efficacy and academic performance.

#### 1.3.4. School Connectedness

Social connectedness is defined as a sense of belonging to a group of people and a feeling of relatedness to them [43]. Social connectedness has been associated with improved psychological and physiological health [44]. On college campuses, increased social connectedness has been reported to contribute toward decreasing the perception of stress [45]. In educational settings, school connectedness or connectedness with peers and the school environment has been related to improvements in learning processes and increased levels of class participation [46].

#### 1.3.5. College Gratitude

Gratitude is a general state of mind in which people are thankful for what they have been blessed with and appreciate the things that benefit their lives [47]. The outcomes of gratitude are usually in the form of a positive emotional response to an external source, when it is of some type of benefit. In terms of the outcomes of gratitude for life experiences, it adds to the quality of life by producing a sense of satisfaction and reducing feelings of depression and anxiety [48,49]. Gratitude has positive implications for academic retention, persistence, and success for college students [50]. College gratitude has been reported as the biggest contributor to the overall happiness of college students [51].

Following Ravitch and Riggan [52], this study used the college student covitality conceptual framework as a guide for aligning the literature, research design, and methodology for the proposed work. This conceptual framework allowed the researchers to situate the study on solid theoretical foundations and in a higher-educational context. The four constructs (i.e., academic satisfaction, academic efficacy, school connectedness, and college gratitude) are significant to the overall study design. They are used as guiding principles for the development of the research questions, data collection instruments (both quantitative and qualitative), and data analysis. This conceptual framework informed the researchers in deploying methods to answer the proposed research questions. In sum, it acted as the glue to keep various parts of the research study intact and interacting purposefully.

## 2. Materials and Methods

This study was conducted during the fall 2021 semester in the college of engineering at a land grant public institution located in the western United States. In this study, we followed a qualitative research approach [53] and purposefully selected and interviewed eight undergraduate engineering students within the college of engineering to understand their positive experiences that contribute to their overall wellbeing. An abundance of literary sources (articles, books, and guides) maintains that between 5 and 50 interviews can be an appropriate participant number for a research study [54]. Data collected from these interviews were then thematically analyzed, as suggested by Creswell and Poth [53]. In the following sections, details of the steps taken to ensure the quality of the interviews and their outcomes and positive implications for the following mixed-method study are described.

### 2.1. Development of Interview Questions

Four interview questions were carefully developed to answer the research question. Special considerations were given to the fitness and relatedness of the interview questions to the conceptual framework being used for this study. The standardized items of the CSSWQ, which correspond to one of the four constructs in the conceptual framework, were used as a guide to the development of the interview questions (see Appendix A). Therefore, the conceptual model provided grounding for both this prior study and the main mixed-methods studies.

One interview question was developed for each of the four constructs that comprise the conceptual model: academic satisfaction, academic efficacy, school connectedness, and college gratitude. While the CSSWQ instrument items are general in nature, the interview questions were developed to contextualize the experiences lived by engineering students at the college of engineering and generate detailed information about the condition of SWB of undergraduate engineering students within and across each of the four constructs. 

**Question 1:** Academic satisfaction: Can you please describe your level of satisfaction with your overall academic experience and coursework progression in the college of engineering?

**Question 2:** Academic efficacy: How do you describe yourself in terms of carrying out academic tasks?

**Question 3:** School connectedness: Overall, do you think the college of engineering allows you to be who you are without being judged? Why or why not?

**Question 4:** College gratitude: In what ways are you appreciative of the support and opportunities you receive within the college of engineering?

### 2.2. Conducting the Semis-Structured Interviews

Semi-structured interviews are frequently used in qualitative research to generate detailed and contextualized participant data using a flexible protocol that enables “dialogue” to occur between the researcher and interviewer. Semi-structured interview protocols often begin with periodic pre-formulated questions that can be supplemented with follow-up questions whenever necessary [55]. An interview protocol (provided in Appendix B) was prepared for the study to ensure maximum utilization of time and resources during the semi-structured interviews and guide the overall interviewing process. Interviews were carried out via the Zoom desktop application. To ensure the privacy and confidentiality of the participants, only the audio recording feature of Zoom was used. 

### 2.3. Participant Selection

After receiving approval from the Institutional Review Board (IRB), undergraduate engineering students were recruited for participation via two strategies to ensure maximum engagement. First, an email was sent to professors to request them to post a recruitment announcement on their Canvas course websites. The announcements asked students to complete an online screening survey in Qualtrics to be considered for a research interview, where they would be eligible to win a $25 Amazon Gift card if selected from a randomized drawing. Second, flyers were also posted on notice boards and other available spaces in the main building of the college of engineering and its adjacent buildings for the same purpose. 

Responses to the online screening survey were used to purposively select participants. The screening survey asked participants to respond to 13 demographic questions, to generate adequate demographic data to use to maximize the diversity of interview participants. For example, students were asked to provide information relating to their age, engineering major, ethnicity, gender, and year of study as well as if they were first-generation college students and if they were traditional or non-traditional students. If students indicated that neither of their biological parents completed a 4-year college degree, they were considered first-generation college students [56]. The seven characteristics identified by Horn [57] were used to determine if students were traditional minimally/moderately/highly nontraditional: (1) their high school or equivalent credential, (2) if they started college within the first 12 months of their graduation from high school, (3) their eligibility for any financial assistance, (4) if they were single parents, (5) if they had any dependents other than a spouse, (6) their enrollment status, and (7) their employment status. Following Horn [57], students were considered to be traditional if they possessed none of these characteristics, minimally non-traditional if they possessed one of these characteristics, moderately non-traditional if they possessed two or three of these characteristics, and highly non-traditional if they possessed four or more of these characteristics. 

Attempts were made to interview a pool of students that was representative of the overall demographic composition of the college of engineering at the host institution. The demographic characteristics of the eight interview participants are shown in Table 1. These sample demographics are comparable to the overall demographic composition of the student population in the college of engineering. According to the university’s Office of Analysis, Assessment and Accreditation, of the total undergraduate enrolment currently (*n* = 1906) in the college of engineering, approximately 91% are White, while 16% are women [58]. As shown in Table 1, eight undergraduate engineering students (3 women, 5 men, 1 Asian White, 2 Latinx White, and 5 White) were interviewed for the study. Therefore, the selected participant sample was more diverse than the general student population. Additionally, four of these participants were first-generation students, while three were moderately traditional. Two participants, one each from the Bioengineering and Electrical and Computer Engineering departments agreed to participate in an interview as a result of contact based on responses to the screening survey. Other departments, i.e., Civil and Environmental Engineering and Mechanical and Aerospace, were represented by three participants each. 

### 2.4. Qualitative Analysis of the Interview Data

**Figure 1:**
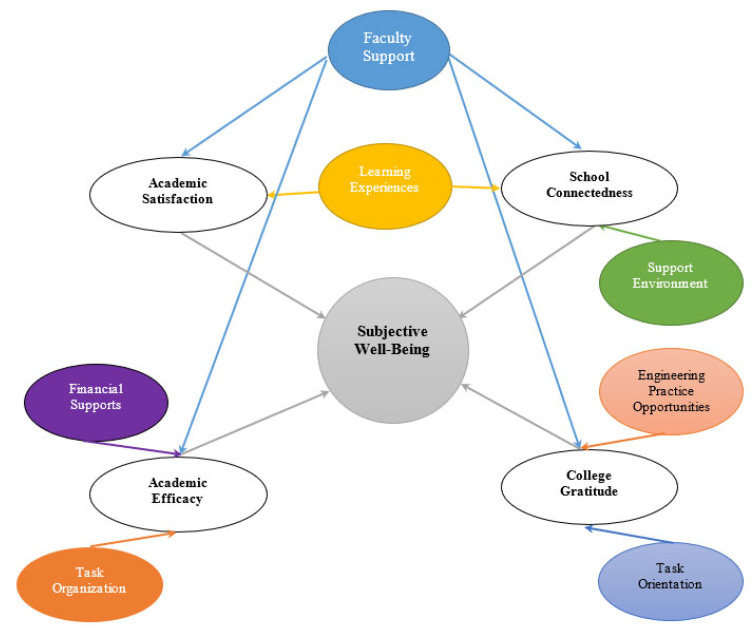
Seven factors model: seven factors perceived to contribute to cumulative SWB via four constructs.

Before analysis, interview transcripts were de-identified after their Zoom-generated audio recordings were transcribed using Trint, an online audio transcription software [59], and verified. Transcripts of participant interviews were inductively coded using thematic analysis techniques. The qualitative data analysis process was iterative and cyclical and involved coding, categorizing, and theming, as described by Saldaña [60]. An initial cycle of reading and rereading the respondents’ interview data was carried out to develop descriptive codes. These codes, with their definitions, were transferred to a coding table. Interview data, in the form of individual excerpts, were then line-by-line coded and grouped into categories according to these descriptive codes. After three passes of categorizing data into subsequent superordinate categories, 11 themes corresponding to the four conceptualized constructs were formed based on the superordinate categories (Table 2). These 11 themes were then re-grouped and conceptualized into 7 perceived factors (Figure 1) for clear mapping, understanding, and explanation. Table 2 provides an overview of the constructs, the superordinate categories, the themes, and their corresponding conceptualized factors. 

Two coders, each with approximately equal responsibility, were involved in the coding process. The first coder was a researcher, while the second coder was a peer graduate student acting as a graduate research assistant in the same department (i.e., the department of engineering education) and in their last semester. An equal amount of text was coded/categorized/themed by each coder in each cycle. During each coding cycle, each graduate student verified the codes/categories/themes and provided feedback to the other before proceeding to the next cycle. Since both graduate students worked in the same facility in the college of engineering, they met in person to discuss and resolve any disagreements or discard codes. A consensus-based coding process was followed. An inter-coder reliability percentage was not calculated during the coding process because a) the graduate students worked toward full consensus rather than a percentage consensus and b) numerical estimations of inter-coder reliability, due to their strongly positivistic nature, may not yield consistent or meaningful results for studies relying on non-positivistic epistemologies [61].

## 3. Findings

RQ: What and how do different factors positively contribute to the SWB of undergraduate engineering students?

To answer the study RQ, qualitative thematic analysis of the interview data revealed seven specific factors (Figure 1) that contributed to undergraduate cumulative SWB via the four SWB constructs of academic satisfaction, academic efficacy, school connectedness, and college gratitude. The seven factors that resulted from data analysis include faculty support, learning experiences, support environment, financial support, opportunities for hands-on engineering practice, opportunities for task organization, and the objective task orientation of the engineering activities provided. 

Figure 1 shows the path through which the perceived seven factors contribute to the cumulative SWB of undergraduate engineering students through the four constructs. For example, faculty support maps to SWB through all four constructs. Learning experiences map to SWB via academic satisfaction and school connectedness, while the support environment also maps through school connectedness. Two factors, financial support and task organization, map to SWB through academic efficacy. Two additional factors, engineering practice opportunities and task orientation, map to SWB through college gratitude.

The following section explains each of these factors and how they connect to one of the four constructs of the conceptual model.

### 3.1. Faculty Support (All Four SWB Constructs)

Engineering undergraduate participants perceived faculty support to be the most important factor in their cumulative SWB. Faculty support was reported to be vital for student wellbeing in the data for all four questions/constructs, as shown in Figure 1. Faculty contribute toward academic satisfaction by enabling students to understand course material inside the class and outside of the class during office hours. The competence of the faculty in delivering course material was also a factor that created a sense of satisfaction. Faculty availability, competence, and practical research experiences positively affected academic efficacy, as they helped students confidently understand their course material and efficiently perform their academic tasks. One of the participants reflected on their satisfaction with the faculty’s support toward their attainment of confidence in achieving academic goals as follows:

*“It seems like all of the professors in the college of engineering are so involved in their own research. They implement it in the classroom and then just make it so we learn quicker I would say. It’s also more of an applied situation. They give a lot of examples of what the specific topic being taught applies to in the industry. So I would say overall highly satisfied.”*

(Participant 8).

Participants also reported that faculty contribute highly toward enabling a class culture, wherein students do not feel judged and can maintain a positive attitude toward each other. All female participants were of the view that there was no gender bias in the college of engineering. One female participant expressed such feelings by saying:

*“I feel like engineering in general, it just happens to be very male-centric. And that’s an issue with just the students, like the population of those classes, and not the teachers I would say teachers do a good job of making it available for everyone and on equal footing for everyone.”*

(Participant 2).

These perspectives of gender inclusion ultimately create a positive feeling toward the college of engineering overall and help students connect with the overall college environment. Participants were grateful for all the support they receive from the faculty and perceived it to be a contributor to their academic success. Another student who had participated in undergraduate research at a professor’s lab had the following to say:

*“I have been presented with a lot of opportunities. I talk about this often with my family. My circuits Professor presented me with an opportunity to participate in some of his research at the power electronics lab and the electric vehicle and roadway at the innovation campus and that opened the door to so many opportunities.”*

(Participant 7).

The above quote suggests that participants were very grateful for the undergraduate research opportunities made available to them by the faculty through their funded projects. 

### 3.2. Learning Experiences (Academic Satisfaction and School Connectedness) 

Academic satisfaction was manifested by learning experiences in addition to faculty support. Learning new material was considered to be an interesting experience by many participants. Participants reported working in collaboration with each other to learn their course material and complete tasks to succeed. Faculty also seem to do their best in enhancing learning experiences, as discussed earlier in detail. The contribution of learning experiences to academic satisfaction through the help of the faculty was detailed by a first-generation student participant as follows:

*“I really think the biggest one, I can say, just the academic help I got from my professors for just being available at all times, if you email them or talk to them, very, very, very helpful, you know. Especially for me because when I came in I didn’t have a background in engineering, you know, none of my parents were engineers or anybody in the family really. So coming into it was a whole new experience for me and they are all just very helpful answering questions and helping me through it so yeah.”*

(Participant 6)

Another participant had the following to say about their learning experiences:

*“I feel like now, now that I am in my senior year and even junior year I feel like the students have really worked together like we’ve collaborated a lot to succeed together in a class.”*

(Participant 7)

As can be observed in the quotes above, collaborative and supportive learning experiences provided a means for participants to connect with peers and faculty and ultimately feel connected with the college of engineering and not be judged in any way due to their gender or personal views. 

### 3.3. Support Environment (School Connectedness) 

The overall support system that was available in the college of engineering helped students connect with the college and have positive feelings about it. It was reported that the faculty and staff were very helpful and were invested in solving the academic and administrative issues that the students face. Peers helped each other, while working in groups without judging each other. The college was reported to have arranged workshops at the tutoring center to promote professionalism in students. According to study participants, the overall support environment characterized by the possible support from peers, faculty, and administration exists and enables them to create a bond with their college and feel a strong connection to it. Apart from the faculty support explained earlier and staff support in the form of resolving administration issues for students relating to course scheduling, tuition, etc., participants seemed specifically impressed with the non-judgmental environment at the college of engineering. This helped and motivated them to have established a positive connection with their college and ultimately contributed positively to their wellbeing. Following are quotes from two of the three female participants.

*“I don’t think that I do feel judged actually. I feel like my male colleagues, they all respect me. I feel respected. I don’t feel like any of them think that they’re smarter or better.”*

(Participant 7).

*“I feel like lots of the students in the college of engineering have the same kind of mindset if that makes sense. We are not all the same right, but I feel like there’s a pretty general mentality and open-mindedness, I think, within the college of engineering. So I am comfortable being who I am among my peer students.”*

(Participant 4).

Participant 4, a female engineering undergraduate, attributed these feelings of inclusion, respect, and non-judgmental treatment to the recent efforts carried out by the government and educational institutions to ensure female inclusion in science, technology, engineering, and mathematics (STEM). In her words:

*“The fact that I am a woman in engineering, I feel like I have lots of opportunities to do better. Or just lots of support and opportunities, because everyone wants women in STEM and wants women to have these kinds of careers. So, I feel like there are lots of opportunities with that … So I just feel like there’s lots of support and opportunities.”*

This may be suggestive that the ongoing efforts taken regarding women’s inclusion in STEM fields may be yielding fruits.

### 3.4. Financial Support (Academic Efficacy)

Participants reported that financial support from a variety of sources was related to their academic efficacy or the belief that they can achieve their academic goals and perform well. Specifically, financial support enabled students to focus on their academics instead of worrying about needing to earn money. Financial support from parents in the form of cash or familial housing (without paying any rent), different types of scholarships, and job opportunities at the college of engineering were some examples of financial support that were useful for supporting participants’ academic efficacy. An interesting comment was made by one of the undergraduate engineering students about how helpful it is toward academic efficacy “to be a local” in financial terms. They were of the view that saving on rent by living at home helps regarding working less compared to their peers and, thus, having more time to focus on schoolwork.

*“I think that’s due to the fact that I don’t have to work as much as other people I have more availability when it comes to doing schoolwork. You know I am here, I am from Logan. So like I am a resident, it’s a little bit cheaper it’s not as expensive for me.”*

(Participant 1).

Financial support helped students focus on achieving their academic goals with less concern about working more hours to support their studies. 

### 3.5. Task Organization (Academic Efficacy)

Having the ability and know-how to be organized and responsible helped participants in achieving academic efficacy. Task organization (which resulted from self-organized behavior) for keeping tight schedules, planning tasks, completing tasks/assignments on time without waiting for deadlines, and breaking larger tasks into small manageable chunks. Several strategies were employed to approach academic tasks in an organized manner. For example, as can be seen in the following quote, keeping separate minders for different classes helped regarding self-organization to efficiently perform academic tasks.

*“I would consider myself pretty organized. Like I have separate minders for different classes. They have been labeled like for watching which ones they are.”*

(Participant 5).

One of the participants elaborated on their strategy for successful task completion as follows:

*“For example, if I had an assignment due tomorrow morning for one of my classes and it was assigned yesterday. So instead of waiting until this afternoon or this evening to do it, I finished the lectures for that day and began working immediately on that assignment. Finishing in advance, if possible, is how I try to approach those tasks.”*

(Participant 3).

Finishing academic tasks in advance and not waiting until the last minute was another strategy used by some participants to build confidence in their abilities to perform well academically.

### 3.6. Hands-On Engineering Practice Opportunities (College Gratitude)

Participants were predominantly grateful for the hands-on opportunities available in the college of engineering. These opportunities helped students learn the practical implications of their theoretical course material. There are several labs available corresponding to different engineering courses. The Ideas Lab, a facility in the college of engineering that is open to all students to try out engineering experiments of their liking, was also appreciated by some participants. Undergraduate research through faculty (discussed earlier) and clubs were also among the hands-on practical opportunities that participants were grateful for. When asked what specifically about the hands-on experiences they were grateful for, one participant expressed their views as follows:

*“I think that one of my really favorite things is to actually get like hands-on experience and not just learning through PowerPoints or like lectures. You know I really like actually going into the lab and actually performing experiments.”*

(Participant 7).

Hands-on experiences were a source of fulfillment for the participants who wanted to try the practical phenomenon behind their theoretical class work. 

### 3.7. Task Orientation (College Gratitude)

One of the reasons for college gratitude was the objective nature of engineering as a field. This objective nature of engineering enabled an environment where students can be more task-oriented. By being task-oriented, we want to insinuate that such people, when working in collaboration with others are more interested in how they can perform the tasks at hand to productively achieve their ultimate goals, with less attention to social relations among the group members. In other words, we want to make a clear distinction between task-oriented and relationship-oriented personality types [62] Students were grateful for and gave more importance to the mutual academic benefits of their relations with their peers, which helped in completing tasks and achieving academic goals rather than in creating social bonds. The following two quotes are good examples of appreciation for task focus and objectivity in engineering educational contexts.

*“I would hope that I am being a part of the environment that allows people to be who they are. Obviously, we have all our different ideals. Social views and personal opinions are different. But I feel like in the college of engineering, what matters most among students is how well we can accomplish our tasks and how we can work with others to achieve those same results.”*

(Participant 3).

*“Whereas in other social spaces, I think you know what you believe, or you know how you choose to portray yourself may have an impact. But here (at the college of engineering) it’s all just numbers and graphs so there’s no real like judgment based on your opinion because you know everything is very, very factual very logical based”*

(Participant 1)

The objective nature of the environment was appreciated by the participants and was considered a motivating force toward success.

## 4. Discussion

Faculty support emerged to be the most important factor to contribute to the SWB of engineering undergraduates. Faculty are identified as the front line of support for students’ mental health and wellbeing needs [63]. Their role has been identified to be among the most important contributors to the wellbeing of students by multiple studies [63,64,65], with the quality of the teacher–student relationship being crucial for a student’s wellbeing [64]. The findings and the corresponding quotes from our research align with the findings from other research, which emphasize the importance of faculty in the wellbeing of undergraduate engineering students. Especially during COVID-19, when a transition was made to emergency remote teaching, faculty support was perceived as vital to their wellbeing by engineering undergraduates, as it helped reduce the students’ stress and feelings of uncertainty [9].

A positive perception of learning experiences was an important ingredient to ensure the wellbeing of engineering undergraduates in our study. In the other literature, we also noticed complementing trends that support the importance of student learning experiences. For example, Stanton and colleagues suggested an enhancement of the learning experiences in an academic environment to positively contribute to the wellbeing of the involved students [66]. Our findings are also consistent with research that links learning experiences and wellbeing via a positive perception of school connectedness. For example, learning experiences have been reported to influence school connectedness as an aspect of students’ wellbeing by contributing to the overall health-promoting measures in educational settings [67].

Research shows that the goal of students’ wellbeing can be achieved only if there is an efficient support system in place that connects students with the institutional actors including administration, faculty, and peers [68]. Our research showed that not only male participants but also female participants considered their college environment to be supportive of and contributing to their wellbeing. The female participants in this study reported positively about their experiences within the college of engineering, despite the outer literature reporting that female engineering undergraduates frequently report negative perceptions about their inclusion in the college of engineering study environments [69] at other institutions.

The research literature has identified a strong correlation between the socioeconomic status where students originate from and academic achievements [70]. Students who receive need-based aid, as compared to peers who do not receive need-based aid, are reported to have higher levels of academic achievement [71]. The other literature is abundant in support of a positive correlation between student financial support and the ability to perform well academically [72,73,74]. These literary sources align with our findings that financial support in different forms contributed positively toward the study participants’ academic efficacy and, hence, added to their cumulative wellbeing. Academic efficacy, in the form of possession of the personality traits of high self-organization, better time management, and, hence, efficient task planning, has been associated with self-control and better coping with life stressors [75]. 

Engineering is a demanding field with practical hands-on engineering practice experience being an integral part of it. Complementing our findings about the importance of hands-on experiences for student wellbeing, such experiences have also been reported to receive a staggering positive response from engineering students in other research and have been deemed as an “integral part of engineering curriculum reform” in the past [76]. This emphasis on hands-on experience and the practical implications of their knowledge may be a reason dictating engineering students to have a positivistic mindset and think objectively. In their professional careers, engineering practitioners are expected to produce solutions to improve human conditions, but the curricula may not be suitable to promote a human-centered philosophy [77]. This may be the reason that our findings point toward a mentality possessed by the participants who are more interested in job completion compared to any consideration for creating social bonds with the people with whom the job is completed. Curricular and systematic changes might be needed to advocate for the importance of human-centered ideals in engineering classrooms, as the effects go well beyond it and are felt in society in the long run, with engineers being such an integral part of society.

## 5. Limitations

The primary limitation of this study is related to the participant sample. As is common in qualitative studies, this study did not produce results generalizable to other contexts due to the limited number (8) of study participants [78]. We did attempt to produce trustworthy and transferable results. Our study does not make broader claims, but our findings are transferable, as the target population may be able to relate them to their personal experiences. 

A second limitation of this study is self-selection bias. Self-selection bias occurs when participants have the choice to participate in the study or not and may produce biased data that are not well representative of the target population [79]. 

## 6. Implications

Our study has specific implications for students and educational institutions. We studied conditions and processes, as perceived by undergraduate engineering students, that contribute to their wellbeing and can help them flourish as a part of a college of engineering. Next, we identified seven factors that contribute to their wellbeing in four constructs (academic satisfaction, academic efficacy, school connectedness, and college gratitude) under the covitality principle. For students to achieve wellbeing, they can focus on working on the identified seven factors and use them to their advantage. We acknowledge they may not always be in sufficient control to do so, as institutional barriers do exist that may hinder their efforts. For example, students might need (1) extra faculty office hours and remote communication access, (2) access to well-equipped labs and maker spaces, (3) more undergraduate research and internship opportunities, or (4) a variety of mechanisms for financial support. This is where institutions need to play a constructive role and take steps to create a college support environment where students are assisted and provided with opportunities that contribute to their overall wellbeing. 

A general implication of this study is to inform the engineering education community about the factors that can be used to guide the development of course curriculum or counseling interventions to help prevent or reduce mental health issues in engineering by supporting students’ SWB. Positive psychology research is used to develop interventions that can be utilized to prevent mental health problems [80]. This present research and other research in similar directions, rooted in positive psychology, can allow policy makers in engineering education to develop strategies to improve the wellbeing of their students before they end up with severe mental health problems. 

## 7. Future Work

As mentioned earlier, the present study was conducted in advance of a larger mixed-methods research project. The survey for the mixed-methods study will consist of three parts, i.e., (1) demographic questions, (2) CSSWQ questionnaire items, and (3) open-ended questions. The quantitative part of the mixed-methods study will consist of the CSSWQ. The qualitative part of the mixed-methods study will consist of open-ended questions (Table 3) developed from this study’s interviews. 

Therefore, both the quantitative and qualitative parts of the mixed-methods study will stem from the same conceptual framework and are expected to harmonize and yield valuable, mixed results. 

## 8. Conclusions

This exploratory study sets out to better understand the collegiate experiences of undergraduate engineering students that contribute to their SWB through four constructs: academic satisfaction, school connectedness, academic efficacy, and college gratitude under the covitality principle. A thematic analysis of the interview data generated from eight engineering students resulted in the conceptualization of a seven-factor model (Figure 1). These seven factors are manifested in the four constructs and are faculty support, learning experiences, support environment, financial support, task organization, engineering practice opportunities, and task orientation. Outcomes from this research have positive implications for the SWB of undergraduate engineering students, with support from their educational institutions. 

## Figures and Tables

**Table 1:** Interview participants’ demographics.

Participant ID	Engineering Major	Gender	Ethnicity	Year of Study	First Generation	Traditional/ Non-Traditional
Participant 1	Mechanical and Aerospace Engineering	Male	Latinx White	Third Year	Yes	Minimally Non-Traditional
Participant 2	Mechanical and Aerospace Engineering	Female	White	Third Year	Yes	Traditional
Participant 3	Civil and Environmental Engineering	Male	Latinx White	Second Year	No	Minimally Non-Traditional
Participant 4	Civil and Environmental Engineering	Female	White	Second Year	Yes	Moderately Non-Traditional
Participant 5	Mechanical and Aerospace Engineering	Male	White	First Year	No	Minimally Non-Traditional
Participant 6	Civil and Environmental Engineering	Male	White	Fourth Year	Yes	Moderately Non-Traditional
Participant 7	Bioengineering	Female	Asian White	Fourth Year	No	Moderately Non-Traditional
Participant 8	Electrical and Computer Engineering	Male	White	Third Year	No	Minimally Non-Traditional

**Table 2:** Constructs, superordinate categories, themes, and their corresponding conceptualized factors.

Constructs	Superordinate Categories from Data	Themes/Factors
Academic Satisfaction	Satisfied because of the professor’s willingness to help and support students Satisfied because the professors are competent in their subjects	Faculty is academic competence and willing to help students is satisfactory/Faculty Support
	Satisfaction due to learning experiences Satisfaction with “weed-out” courses, as they are necessary for efficient learning Satisfied with hands-on experiences tailored to courses Satisfied with the structure of the curriculum, as courses build upon each other	Satisfied with opportunities available for efficient learning/Learning Experiences
Academic Efficacy	Supported by professors to understand and complete academic tasks Professor’s availability within the class and office hours to discuss course material and academic tasks	Faculty support efficient completion of academic tasks/Faculty Support
	Financial support helped focus efficiently on completing academic tasks Types of financial support: Scholarships Financial support from home Part-time jobs at the college of engineering	Financial support ensures focus on academics/Financial Support
	Scheduling academic tasks Planning for each task Properly understanding and doing academic tasks, while dividing them into manageable chunks Doing assignments in advance of deadlines Acting as organized students	Proper organization of tasks enables their efficient completion/Task Organization
School Connectedness	Professors contribute highly to maintaining a positive environment at the college of engineering Professors provide equal opportunities to all	Faculty efforts contribute to positive attitudes toward college/Faculty Support
	Students help each other learn course material Students collaborate to conduct academic tasks such as assignments and exam preparation	Available learning opportunities positively relate to the college/Learning Experiences
	Students can be who they are Students are friendly with their peers Professors provide equal opportunity to all their students Non-judgmental college environment overall No gender-based judgment College staff support students when they need help	The college environment is non-judgmental and supportive/Support Environment
College Gratitude	Appreciate professors making themselves available in person and via email Professors do their best to enable their students to succeed	Grateful for faculty contributions to student success/Faculty Support
	Hand-on experiences are available to tailor theory and practice (e.g., labs) Undergraduate research opportunities available Awareness and participation in internships facilitated by the college of engineering (through clubs) Awareness and participation in student clubs	Abundant hands-on experiences available to tailor theory to practice/Engineering Practice Opportunities
	Grateful for the objective nature of engineering as a discipline in the college of engineering, with minimal judgment based on personal bias Grateful for the goal-focused mentality of peers	Peers have an objective mindset supporting a focus on academic goals/Task Orientation

**Table 3:** Open-ended survey questions developed from the study data.

S. No.	Factor(s)/Construct(s)	Open-Ended Question
1	Faculty support/All constructs	In what ways does the college of engineering faculty contribute to your wellbeing?
2	Learning experiences/Academic satisfaction and school connectedness	How do the learning experiences provided within the college of engineering contribute to your academic satisfaction and/or feelings of connectedness within the college?
3	Financial support/Academic efficacy	How does financial support from different sources enable you to complete your assigned academic tasks successfully?
4	Task organization/Academic efficacy	How do task organization strategies enable you to complete your assigned academic tasks successfully?
5	Support environment/School connectedness	How does the support provided within the college of engineering contribute to your feelings of connectedness within the college?
6	Engineering practice organization and task orientation/College gratitude	How does an environment focused on engineering practice contribute to your feelings of gratitude toward the college of engineering?
7	General	Can you please describe any other factors that contribute to your wellbeing within the college of engineering?

## Data Availability

The data that support the findings of this study are available from the corresponding author upon reasonable request.

## Appendix A

**Table A1:** College student subjective wellbeing questionnaire (CSSWQ) items and their corresponding study interview questions in four domains.

Constructs and Items	Qualitative Questions
**Academic satisfaction** I have had a great academic experience at the college of engineering. I am happy with how I’ve done in my classes. I am satisfied with my academic achievements since coming to the college of engineering. I am pleased with how my college education is going so far.	Can you please describe your level of satisfaction with your overall academic experience and coursework progression in college of engineering?
**Academic efficacy** I am a hard worker in my classes. I am a diligent student. I am an organized and effective student. I study well for my classes.	How do you describe yourself in terms of carrying out academic tasks?
**School connectedness** I feel like a real part of the engineering college. People at this school are friendly to me. I can really be myself at this school. Other students here like me the way I am.	Overall, do you think the college of engineering environment allows you to be who you are without being judged? Why or Why not?
**College gratitude** I am so thankful that I’m getting a college education. I am grateful to the professors and other students who have helped me in class. I feel thankful for the opportunity to learn so many new things. I am grateful for the people who have helped me succeed in college.	In what ways are you appreciative of the support and opportunities you receive within the college of engineering?

## Appendix B

Semi-structured interview protocol

**Pre-Interview**

- Contact participant to remind them of the interview time and share the link to Zoom conference (online meeting).
- Make sure audio recording is working.
- Print out interview notes template and interview protocol.
- Join the video conference (online meeting) at least 10 min prior to scheduled time to set up interview space.

**At the Time of Interview**

This semi-structured interview is aimed at gathering qualitative data related to our research study, “subjective wellbeing of undergraduate engineering students.” The interview is conducted on the Zoom online application and is audio recorded. The following protocol will be followed to ensure the interview is conducted smoothly.

1. **Timing**

Zoom meeting room will open at the agreed time.

The interview will last for about 30 min.

2. **Role of researcher**

Researcher will act as the interviewer.

3. **Conducting the interview**

Reminder #1: Zoom audio recording is on.

Reminder #2: Notes are taken during the interview process.

**Sequence of events:**

**Introduction and Informed Consent**

- Introduction of the researcher.
- Confirm consent form receipt.
- Brief introduction about the project: The purpose of this study is to investigate the status of subjective wellbeing of undergraduate engineering students by interviewing these students and the faculty members who have been teaching undergraduate engineering courses at the college of engineering.
- Reminder: Information from this interview is confidential but not anonymous. Please do not share information shared within the interview outside of this meeting. No names will be used while analyzing, presenting, or publishing data from this interview recording.

**Discussion**

- Participants are encouraged to express their opinions openly and respond to other participants as well.
- The researcher starts the discussion with the first interview question from the list provided at the end of this document.
- The researcher will take notes about the start time and end time of the discussion and keep the discussion within time.
- The researcher may ask follow-up questions as necessary.
- After each question, the researcher summarizes what has been discussed.
- After the summary, the researcher asks participants if they have more comments.

Reminder #3: Allow appropriate time for each question, so the interview is conducted in a timely fashion.

**Closing the interview**

- Participants are asked if they have any other items of discussion or questions/concerns about the study.
- Participants are reminded that data from this interview will be qualitatively analyzed and published.
- All participants are thanked for their participation.

**Semi-structured interview questions**

- Can you please describe your level of satisfaction with your overall academic experience and coursework progression in the college of engineering?
- How do you describe yourself in terms of carrying out academic tasks?
- Overall, do you think the college of engineering environment allows you to be who you are without being judged? Why or why not?
- In what ways are you appreciative of the support and opportunities you receive within the college of engineering?

Note: Responses to the above questions may lead to follow-up questions necessary to understand participants’ experiences completely and may provide valuable insights into the issue under study. Follow-up questions may be asked as they emerge during the course of the interview. However, all the questions will only be related to the topic under investigation, not regarding any private information. Moreover, the interviewees will have a choice to skip any questions they do not want to answer at any stage of the interview.
